# Characteristics of *CARMA1-BCL10-MALT1-A20-NF-κB* expression in T cell-acute lymphocytic leukemia

**DOI:** 10.1186/s40001-014-0062-8

**Published:** 2014-11-11

**Authors:** Yu Ma, Ziwei Liao, Yi Xu, Ziyun Zhong, Xu Wang, Fan Zhang, Shaohua Chen, Lijian Yang, Gengxin Luo, Xin Huang, Suming Huang, Xiuli Wu, Yangqiu Li

**Affiliations:** Institute of Hematology, Jinan University, Guangzhou, 510632 China; Department of Medicine, Imperial College London, St Mary’s Campus, London, W2 1PG UK; Key Laboratory for Regenerative Medicine of Ministry of Education, Jinan University, Guangzhou, 510632 China; Department of Hematology, Guangdong General Hospital (Guangdong Academy of Medical Sciences), Guangzhou, 510080 People’s Republic of China; Department of Biochemistry and Molecular Biology, College of Medicine, University of Florida, Gainesville, FL 32610-3633 USA

**Keywords:** *A20*, *CARMA1-BCL10-MALT1*, T-cell acute lymphoblastic leukemia

## Abstract

**Background:**

Knowledge of the oncogenic signaling pathways of T-cell acute lymphoblastic leukemia (T-ALL) remains limited. Constitutive aberrant activation of the nuclear factor kappa B (NF-κB) signaling pathway has been detected in various lymphoid malignancies and plays a key role in the development of these carcinomas. The zinc finger-containing protein, A20, is a central regulator of multiple NF-κB-activating signaling cascades. A20 is frequently inactivated by deletions and/or mutations in several B-and T-cell lymphoma subtypes. However, few *A20* mutations and polymorphisms have been reported in T-ALL. Thus, it is of interest to analyze the expression characteristics of *A20* and its regulating factors, including upstream regulators and the CBM complex, which includes *CARMA1*, *BCL10*, and *MALT1*.

**Methods:**

The expression levels of *CARMA1*, *BCL10*, *MALT1*, *A20*, and *NF-κB* were detected in peripheral blood mononuclear cells (PBMCs) from 21 patients with newly diagnosed T-ALL using real-time PCR, and correlations between the aberrant expression of these genes in T-ALL was analyzed. Sixteen healthy individuals, including 10 males and 6 females, served as controls.

**Results:**

Significantly lower *A20* expression was found in T-ALL patients (median: 4.853) compared with healthy individuals (median: 8.748; *P* = 0.017), and significantly increased expression levels of *CARMA1* (median: 2.916; *P* = 0.034), *BCL10* (median: 0.285; *P* = 0.033), and *MALT1* (median: 1.201; *P* = 0.010) were found in T-ALL compared with the healthy individuals (median: 1.379, 0.169, and 0.677, respectively). In contrast, overexpression of *NF-κB* (median: 0.714) was found in T-ALL compared with healthy individuals (median: 0.335; *P* = 0.001). A negative correlation between the *MALT1* and *A20* expression levels and a positive correlation between *CARMA1* and *BCL10* were found in T-ALL and healthy individuals. However, no negative correlation was found between *A20* and *NF-κB* and the *MALT1* and *NF-κB* expression level in the T-ALL group.

**Conclusions:**

We characterized the expression of the *CARMA-BCL10-MALT1-A20-NF-κB* pathway genes in T-ALL. Overexpression of *CARMA-BCL10-MALT* in T-ALL may contribute to the constitutive cleavage and inactivation of *A20*, which enhances *NF-κB* signaling and may be related to T-ALL pathogenesis.

## Background

T-cell acute lymphoblastic leukemia (T-ALL), which accounts for 15% of all newly diagnosed ALL cases in children and 20 to 25% of ALL cases in adults, results from clonal malignant T-cell proliferation, is an aggressive malignancy that does not respond well to chemotherapy, and has poorer prognosis than its B-cell counterpart [1-3]. The cellular biology and pathogenesis of T-ALL are relatively complex, and these might be related to the different original malignant T-cell clone, e.g., T-ALL cases with two malignant T-cell clones or a mono-malignant T-cell clone have different gene expression patterns [4-6]. It has also been reported that the acute and lymphoma subtypes of adult T-cell leukemia/lymphomas are genomically distinct; thus, they may develop tumors via different genetic pathways as suggested by comparative analysis of their genomic profiles [7]. Complex acquired genetic aberrations include chromosomal translocations, and gene rearrangements and mutations, resulting in the abnormal expression of oncogenes such as *Notch*1, *TAL1* (T-cell acute lymphoblastic leukemia 1), and *BCL11B* (B-cell chronic lymphocytic leukemia/lymphoma 11B), which may be associated with advanced disease and resistance to treatment [8-14]. In contrast, abnormal T-cell activation is vital for cellular transformation, and various signaling pathways are triggered by the T-cell receptor that play key roles in controlling T-cell activation. For example, recent findings define MALT1 (mucosa-associated-lymphoid-tissue lymphoma-translocation gene 1) as a protein with proteolytic activity that controls T-cell activation by regulating key molecules in T-cell receptor (TCR)-induced signaling pathways [15]. Moreover, a number of studies have shown that in A20 (tumor necrosis factor-α-induced protein 3; TNFAIP3), which is a nuclear factor kappa B (NF-κB) negative regulator, genetic alterations are frequently found in lymphomas, suggesting that there may be a link between the altered immune response and leukemogenesis [16-23]. Furthermore, it was shown that TCR stimulation induces the recruitment of A20 into a CBM complex containing *CARMA1* (caspase-recruitment domain (CARD) containing membrane-associated guanylate kinase protein 1, also called CARD11), adaptor protein Bcl-10 (B-cell lymphoma 10), and MALT1 (paracaspase mucosa-associated lymphoid tissue lymphoma translocation gene 1), leading to MALT1-mediated A20 processing. MALT1 cleaves human A20 after arginine 439 and impairs its NF-κB-inhibitory function. A20 is a substrate of MALT1, underscoring the importance of MALT1 proteolytic activity in the ‘fine tuning’ of TCR signaling [24].

A20 is frequently inactivated by deletions and/or mutations in several lymphoma subtypes including B- and T-cells [16-22]. Recently, bi- and monoallelic *A20* deletions in a high proportion of Sezary syndrome patients and a biallelic *A20* deletion in the Sezary syndrome-derived cell line SeAx were identified. Furthermore, A20 inhibition activates the NF-κB pathway, thereby increasing the proliferation of normal T-cells [17]. Interestingly, we recently found that there are rare *A20* mutations and polymorphisms in T-ALL [25]. Therefore, it is of interest to analyze the expression characteristics of *A20* and its regulating factors, including upstream components of the CBM complex, which includes *CARMA1*, *BCL10*, and *MALT1* [24,26-28], which is expected to provide new insight in the abnormal molecular regulation on T-cell activation. In this study, we characterized the gene expression pattern of *A20*, as well as the gene expression levels of its upregulating factors CARMA1-BCL10-MALT1 and its target factor NF-κB in T-ALL.

## Methods

### Samples

The samples used in this study were derived from 21 newly diagnosed, untreated patients with T-ALL, including 17 males and 4 females (4 to 66 years old; median age: 23.5 years). Sixteen healthy individuals including 10 males and 6 females (17 to 45 years old; median age: 26 years) served as controls. Peripheral blood mononuclear cells (PBMCs) were isolated from heparinized venous blood by Ficoll-Paque gradient centrifugation. RNA extraction and cDNA synthesis from PBMCs were performed according to the manufacturer’s instructions. All human peripheral blood samples were obtained with consent from the human subjects. All procedures were conducted according to the guidelines of the Medical Ethics Committee of the Health Bureau of Guangdong Province in China, and ethical approval was obtained from the Ethics Committee of the Medical School of Jinan University.

### Quantitative real-time RT-PCR (qRT-PCR)

The sequences of the primers used for *CARMA1*, *BCL-10*, *MALT1*, *A20*, and *NF-κB* gene amplification are listed in Table 1. The expression level of the *CARMA1*, *BCL-10*, *MALT1*, *A20*, *NF-κB*, and β2-microglobulin (*β2M*) genes was determined by SYBR Green I real-time PCR as previously described [4,25,29,30]. The relative amounts of the genes of interest and the *β2M* reference gene were measured in two independent assays. The specific, amplified PCR products were analyzed by melting curve analysis. The data are presented as the relative expression of the genes of interest compared with the internal control gene as determined by the 2(^-∆CT^) method [4,28-30].

**Table 1:** **List of primers used for real-time RT-PCR**

**Primer**	**Sequence**
CARMA1-f	5′-ttgtgggagaatgtggagtgt-3′
CARMA1-r	5′-tgccccttggtatgtagaatg-3′
BCL10-f	5′-cccgctccgcctcctctcctt-3′
BCL10-r	5′-ggcgcttcttccgggtccg-3′
MALT1-f	5′-tcttggctggacagtttgtga-3′
MALT1-r	5′-gctctctgggatgtcgcaa-3′
A20-f	5′-ctgggaccatggcacaactc-3′
A20-r	5′-cggaaggttccatgggattc-3′
NF-κB-f	5′-ccacaagacagaagctgaag-3′
NF-κB-r	5′-agatactatctgtaagtgaacc-3′
β_2_M-f	5′-tacactgaattcacccccac-3′
β_2_M-r	5′-catccaatccaaatgcggca-3′

### Statistical analysis

Two independent-samples Mann-Whitney U tests were performed to compare the median expression level of each gene between patients with T-ALL and control individuals. Spearman correlation and linear regression analyses were used to determine the association between different genes in different groups. A *P* <0.05 was considered statistically significant [29,30].

## Results and discussion

**Figure 1:**
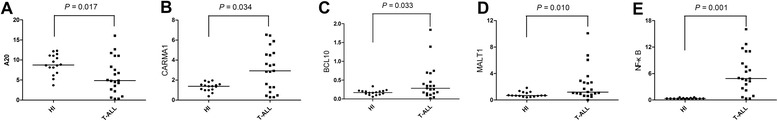
**The expression levels of the (A)** ***A20*** **, (B)** ***CARMA1*** **, (C)** ***BCL10*** **, (D)** ***MALT1*** **, and (E)** ***NF-ΚB*** **genes in healthy individuals and patients with T-ALL.**

Despite significant improvement in our understanding of T-ALL biology and pathogenesis, knowledge of the oncogenic signaling pathways involved in T-ALL remains limited. Constitutive aberrant activation of the NF-κB signaling pathway has been detected in various lymphoid malignancies, and it plays a key role in the development of these tumors. A20 is a central regulator involved in the negative feedback regulation of multiple NF-κB-activating signaling cascades [16,18,31]. Recently, numerous studies showed that A20 is inactivated by deletions and/or mutations in several lymphoma subtypes, including T-cell lymphomas [16-22], and A20 inhibition results in constitutive NF-κB activation in tumor cells. These data indicate that A20 inactivation might play a role in malignant T-cells. Bi- and monoallelic *A20* deletions in a high proportion of Sezary syndrome patients were identified [16]; however, mutations and polymorphisms in A20 rarely occur in T-ALL [24]. Thus, it is of interest to characterize the *A20* expression pattern in T-ALLs containing an *A20* deletion. In this study, we examined the *A20* expression level and found significantly lower *A20* expression in T-ALL patients (median: 4.853) compared with healthy individuals (median: 8.748; *P* = 0.017) (Figure 1A). Thus, we hypothesized that *A20* downregulation may be due to abnormal upstream regulation.

Antigen receptor-mediated NF-κB activation in lymphocytes relies on the formation of a large multi-protein complex containing CARMA1, BCL10, and MALT1 (CBM). MALT1 has proteolytic activity and controls T-cell activation by regulating NF-κB pathways [14,31], and it mediates rapid proteolytic cleavage and A20 inactivation [23]. Studies in MALT1-deficient mice have demonstrated an essential role for MALT1 in TCR- and B-cell receptor-mediated functions [15,28]. The CARMA1-BCL10-MALT1 pathway is pathologically altered in several lymphoma subtypes [32], including activated B-cell-like diffuse large B-cell lymphoma (ABC-DLBCL) [33]. The CARMA1-BCL10-MALT1 pathway also plays a central role in TCR signaling that results in T-cell activation and proliferation [24,26-28,34]. In this study, we attempted to characterized alterations in the CBM genes in T-ALL. We examined the expression levels of the *CARMA1*, *BCL10*, and *MALT1* genes, and significantly increased expression levels of *CARMA1* (median: 2.916; *P* = 0.034), *BCL10* (median: 0.285; *P* = 0.033), and *MALT1* (median: 1.201; *P* = 0.010) were found in T-ALL patients compared with healthy individuals (median: 1.379, 0.169, and 0.677, respectively) (Figure 1B–D). High expression of the CBM genes indicates significantly high leukemic T-cell activation, and high MALT1 expression might mediate A20 downregulation, which was found in the same T-ALL samples. This finding may also partially explain the lower expression level of *A20* in T-ALL. Because CBM mediates TCR-induced NF-κB during T-cell activation, we further analyzed the expression level of *NF-κB*, and as expected, *NF-κB* overexpression (median: 0.714) was found in T-ALL patients compared with healthy controls (median: 0.335; *P* = 0.001) (Figure 1E). Overall, we show that the abnormal expression of CBM and *A20* in T-ALL cells may be related to the abnormal proliferation of malignant T-cells. This result is consistent with the finding that A20 is also a putative tumor suppressor in T-cell malignancies such as Sézary syndrome. In contrast, such abnormal expression characteristics may be considered as biomarkers or target factors in T-ALL.

Overexpression of *CARMA1* was reported in angioimmunoblastic T-cell lymphoma and peripheral T-cell lymphoma, and it was linked to poor prognosis in a report by Fujiwara et al. [35]. Additionally, in a genome profile analysis of aggressive adult T-cell leukemia/lymphoma, *CARMA1* was found to be a potential 7p22 amplification target gene in the lymphoma but not acute subtype. This finding suggests that the acute and lymphoma subtypes are genomically distinct; thus, they may develop tumors via distinct genetic pathways [7]. However, there are few reports of CBM molecular aberrations or dysfunction in T-ALL [4]. In this study, we found that all of the CBM genes were upregulated, resulting in the downregulation of *A20* and upregulation of *NF-κB*, which may be a common characteristic of abnormal proliferation and activation in T-cell malignancies. Therefore, it is suggested that such overexpressed genes may be considered potentially attractive targets for the development of T-ALL therapeutics. It is well known that NF-κB is a target for multiple myeloma therapy via proteasome inhibitors such as bortezomib [36], which was also recently used in combined therapy for T-cell malignancies [37,38]. Moreover, two kinds of small molecule inhibitor for MALT1 have been reported recently. One of them is the phenothiazine derivative mepazine, which has been shown to have promising anticancer properties in subtypes of B-cell lymphoma and could also be used in the treatment of lymphocyte-mediated autoimmune pathologies such as multiple sclerosis [39]. The other one is MI-2 which binds directly to MALT1 and irreversibly suppresses protease function, and displays selective activity against ABC-DLBCL cell lines *in vitro* and xenotransplanted ABC-DLBCL tumors *in vivo*. It would be worthy to investigate the anti-T-ALL effect of such Malt1 inhibitors [40].

**Figure 2:**
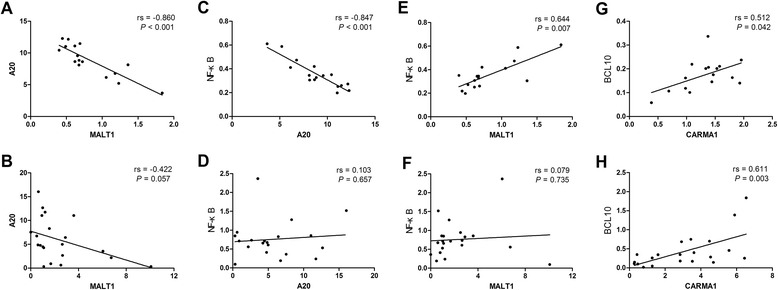
**Correlation analysis of the expression levels of (A)** ***MALT1*** **and** ***A20*** **in healthy individuals (HI), (B)** ***MALT1*** **and** ***A20*** **in T-ALL, (C)** ***A20*** **and** ***NF-κB*** **in HI, (D)** ***A20*** **and** ***NF-κB*** **in T-ALL, (E)** ***MALT1*** **and** ***NF-κB*** **in HI, (F)** ***MALT1*** **and** ***NF-κB*** **in T-ALL, (G)** ***CARMA1*** **and** ***BCL10*** **in HI, and (H)** ***CARMA1*** **and** ***BCL10*** **in T-ALL.**

We further analyzed associations between the expression patterns of the CBM, *A20*, and *NF-κB* genes. A20 is generally cleaved by MALT1; thus, the expression level of MALT1 should be negatively correlated with the A20 and MALT1 expression pattern [41]. We found a negative correlation between the *MALT1* and *A20* expression levels (rs = –0.806, *P* = <0.0001) in the healthy individual (Figure 2A) and T-ALL patient groups (rs = –0.450, *P* = 0.041; Figure 2B) as expected. A negative correlation was found between the *A20* and *NF-κB* expression levels (rs = –0.847, *P* <0.0001; Figure 2C) in healthy individuals, as expected, while there was no significant correlation between the A20 and NF-κB expression levels in T-ALL patients (rs = 0.0208, *P* = 0.929; Figure 2D). Moreover, the negative correlation was lost, and whether this is due to abnormal CMB regulation remains an open question. A positive correlation between the *MALT1* and *NF-κB* expression level was also found in healthy controls (rs = 0.641, *P* = 0.001; Figure 2E), while there was no significance in the correlation between genes in the T-ALL group (rs = 0.193, *P* = 0.402; Figure 2F). Moreover, we found a significant positive correlation between the gene expression levels of *CARMA1* and *BCL10* in healthy individuals (rs = 0.513, *P* = 0.042; Figure 2G) and T-ALL patients (rs = 0.572, *P* = 0.007; Figure 2H). Overall, this result indicates that *MALT1*, *A20*, and *NF-κB* lose their normal expression pattern at the molecular level, and their manner of regulation in T-ALL may be more complex. In our previous studies, we found two T-ALL patients with two malignant Vδ1 and Vδ2 T-cell clones who had poor outcome, and high expression of the *Notch1* and *CARMA-BCL10-MALT1-A20-NF-κB* pathway genes in this biclonal T-ALL patient group compared with a mono-malignant Vα T-cell clone was found [4]. Based on our data, it is worth further investigating whether the different expression patterns of the *CARMA-BCL10-MALT1-A20-NF-κB* pathway genes may be a biomarker for a genomically-distinct subtype of T-ALL or a prognostic biomarker for T-ALL. Increasing new genetic markers for ALL have been found to have prognostic impact [42].

## Conclusions

We first characterized the expression pattern of the *CARMA-BCL10-MALT1-A20-NF-κB* pathway genes and found that overexpression of CBM genes in T-ALL may cause constitutive cleavage and inactivation of A20 to enhance NF-κB signaling, contributing to the pathogenesis of T-ALL. Thus, this pathway may be considered a potentially attractive target for the development of T-ALL therapeutics. However, this finding is based only on results from a limited case analysis and further research involving more samples is needed to determine representative results. Moreover, the change of protein levels of this pathway are needed to confirm this, especially for target therapeutic strategy in T-ALL.

## Abbreviations

ABC-DLBCL: Activated B-cell-like diffuse large B-cell lymphoma
A20: Tumor necrosis factor alpha-induced protein 3 (TNFAIP3)
Bcl-10: B-cell lymphoma 10
BCL11B: B-cell chronic lymphocytic leukemia/lymphoma11B)
β2M: β2-microglobulin
CARMA1: Caspase-recruitment domain (CARD) containing membrane-associated guanylate kinase protein 1 (CARD11)
CBM: CARMA1, BCL10, and MALT1
MALT1: Mucosa-associated-lymphoid-tissue lymphoma-translocation gene 1
NF-κB: Nuclear factor κB
PBMCs: Peripheral blood mononuclear cells
qRT-PCR: Quantitative real-time RT-PCR
T-ALL: T-cell acute lymphoblastic leukemia
TCR: T-cell receptor
